# Effects of Mulching and Nitrogen on Soil Nitrate-N Distribution, Leaching and Nitrogen Use Efficiency of Maize (*Zea mays L*.)

**DOI:** 10.1371/journal.pone.0161612

**Published:** 2016-08-25

**Authors:** Xiukang Wang, Yingying Xing

**Affiliations:** 1 College of Life Science, Yan'an University, Yan'an, Shaanxi, 716000, China; 2 State Key Laboratory of Soil Erosion and Dryland Farming on the Loess Plateau, Institute of Soil and Water Conservation, Chinese Academy of Sciences and Ministry of Water Resources, Yangling, Shaanxi, 712100, China; MJP Rohilkhand University, INDIA

## Abstract

Mulching and nitrogen are critical drivers of crop production for smallholders of the Loess Plateau in China. The purpose of this study was to investigate the effect of mulching and nitrogen fertilizer on the soil water content, soil nitrate-N content and vertical distribution in maize root-zone. The experiment was conducted over two consecutive years and used randomly assigned field plots with three replicates. The six treatments consisted of no fertilizer without plastic film (CK), plastic film mulching with no basal fertilizer and no top dressing (MN_0_), basal fertilizer with no top dressing and no mulching (BN_1_), plastic film mulching and basal fertilizer with no top dressing (MN_1_), basal fertilizer and top dressing with no mulching (BN_2_) and plastic film mulching with basal fertilizer and top dressing (MN_2_). In the top soil layers, the soil water content was a little high in the plastic film mulching than that without mulching. The mean soil water content from 0 to 40 cm without mulching were 3.35% lower than those measured in the corresponding mulching treatments in 31 days after sowing in 2012. The mulching treatment increased the soil nitrate-N content was observed in the 0–40-cm soil layers. The results indicate that high contents of soil nitrate-N were mainly distributed at 0–20-cm at 31 days after sowing in 2012, and the soil nitrate-N concentration in the MN_2_ treatment was 1.58 times higher than that did not receive fertilizer. The MN_2_ treatment greatly increased the soil nitrate-N content in the upper layer of soil (0–40-cm), and the mean soil nitrate-N content was increased nearly 50 mg kg^−1^ at 105 days after sowing compared with CK treatment in 2012. The soil nitrate-N leaching amount in MN_1_ treatment was 28.61% and 39.14% lower than BN_1_ treatment, and the mulch effect attained to 42.55% and 65.27% in MN_2_ lower than BN_2_ in both years. The yield increased with an increase in the basal fertilizer, top dressing and plastic film mulching, and the grain yield increase ranged from 31.41% to 83.61% in two consecutive years. The MN_1_ and MN_2_ treatment is recommended because it increased the grain yield and improved the fertilizer use efficiency, compared with the no-mulching treatment.

## Introduction

Traditionally, gravel mulching was an effective strategy to increase soil temperature and moisture and, therefore, crop production; this method was recently replaced by plastic film mulching with the onset of industrial development [1]. Since the middle of the last century, the advantages of plastic film mulching have been reported that mulching has almost doubled crop production and reduced the harvest time [2–4]. Recently, with the rapid development of modern industry since the 1990s, plastic film mulching has been widely adopted for dryland agriculture in arid and semi-arid areas of China [5–7]. Studies have indicated that mulching was significantly enhanced the soil temperature and soil water content during the seedling stage, which played an important role in maintaining sufficient amounts of heat and water for maize growth [8, 9]. Mulching also has the benefit of improving soil physical conditions, including the protection of topsoil stability [10]. The research have demonstrated that the benefits of plastic mulching result from the adjustment of the soil environment caused by an increase in soil temperature and a reduction in evaporation, weed competition, soil compaction and soil erosion. These changes in the soil environment are good for crop root growth, and the stronger ability of roots, which results in increased absorption of soil water and nutrients [11].

The Loess Plateau has a typical semi-arid monsoon climate, where maize (*Zea mays L*.) is one of the most common crops [12–14]. However, low air temperatures and drought during the early crop growth stage in the spring which may result in poor crop yield. Plastic film mulching was significantly increasing the maize yield in this region [6, 15, 16], and there was two major reasons. First, plastic film mulching reduces soil evaporation by intercepting the steam that is released when water moves from deeper soil layers to the topsoil by capillarity and maintains the stability of the topsoil water content, which increases crop transpiration [17]. Second, plastic film mulching increases the soil temperature as the greenhouse effect, which may absorbs solar radiation above the mulching and reduces heat loss, improving crop production [18, 19].

Increased yield in response to plastic film mulching not only results in improved soil water content and increased soil temperature but also directly changes soil microbial environment and fertility. Maize is one of the high demands for nitrogen crops, and the nitrogen application levels significantly affect grain yield and biomass yield [20, 21]. Particularly, when the nitrogen fertilizer input in excess of the crop requirements, the soil nitrogen accumulation and leaching would happen in the soil profile [22–25]. The research also reported that overuse of chemical nitrogen fertilizers has result in reduced nitrogen use efficiency and considerable nitrogen leaching into groundwater nationwide [26]. Usually this is a very exclusive case in Northwest China, where about 48% of the wheat and 39% of the maize yield were produced by stepped-up cropping [27]. Furthermore, the high rates of nitrogen fertilizer and irrigation amount, which may increase in nitrogen leaching and leading to over-consumption of fresh water resources [27–29].

But above all, the optimum nitrogen application rate in a plastic film mulching ridge-furrow system needs to be determined for high maize yields and potential environmental benefits. The proper nitrogen fertilizer application to meet the crops needs is the key to increase crop yield, nitrogen use efficiency and decrease nitrogen leaching. This requires knowledge of crop nitrogen demand and the amount of available nitrogen offered from the soil through mineralization process. This study evaluated the feasibility of using alternative field management practices to contribute towards food security and sustainable agriculture. Therefore, the aim of the study was to (1) evaluate the effect of mulching and nitrogen fertilizer on the vertical distribution of soil water contents and soil-N content, (2) investigate the soil nitrate-N accumulation, leaching and nitrogen fertilizer use efficiency of maize in Loess Plateau of Northwest China.

## Materials and Methods

### Experimental site

The authority responsible for Changwu Experimental Station who issued the permission for each location, and the field is not privately owned field. We confirmed that the field studies did not involve endangered or protected species.

The field experiment was conducted at the Changwu Experimental Station (35°12´N, 107°40´E and altitude 1206 m) on the Loess Plateau in Changwu county of Shaanxi Province, China. The climate is temperate semi-arid with a mean annual air temperature of 9.1±2.3°C, a mean monthly maximum temperature of 22°C (July) and a mean monthly minimum temperature of –7°C (January). The average annual sunshine duration is 2230 h with more than 171 frost-free days. The mean annual precipitation from 1990 to 2012 was 571±74 mm, of which approximately 55% fell during the growing season between July and September. The rainfall during the experimental period was measured using an automatic weather station (Changwu experimental station meteorological observatory, WS-STD1, England) at the experimental site. The sum of rainfall during the whole growth period of maize was 396 mm in 2012 and 374 mm in 2013, and this accounted for 71.1% and 68.9% of the annual rainfall, respectively. According to the USDA textural classification system, the soil has a silty loam texture, which is derived from loess with a deep and even soil profile. Soil sample was dried at room temperature (75°C) in the laboratory to a constant weight and sieved (2 mm) to eliminate coarse soil particles. Soil acidity (pH) was measured in an aqueous soil extract in de-ionized water (1:2.5 soils: water). Bulk density was measured by the core method, using cores that measured 3 cm in diameter, 10 cm in length, and 70.68 cm^3^ in volume. Field capacity at 33 kPa was determined using a pressure-membrane extraction apparatus. Soil organic matter was determined using the Walkley-Black method [30]. The physicochemical properties of the soil profile were determined in April 2012 and 2013 (Table 1).

**Table 1:** Major soil physicochemical characteristics of the experimental site.

Years	Soil depth (cm)	Bulk density (g cm^−3^)	Organic matter (g kg^−1^)	Total C (g kg^−1^)	Total N (g kg^−1^)	NH_4_^+^−N (mg kg^−1^)	NO_3_^-^−N (mg kg^−1^)	Avail. P (mg kg^−1^)	Avail. K (mg kg^−1^)
2012	0―30	1.23	12.20	15.80	0.67	3.80	10.56	36.5	130.4
	30―60	1.32	10.10	14.70	0.48	2.70	4.78	24.5	141.7
	60―100	1.38	8.70	13.90	0.17	1.90	1.53	15.6	117.8
2013	0―30	1.25	11.70	17.20	0.59	4.20	8.68	42.3	148.4
	30―60	1.37	9.40	15.50	0.37	3.10	4.02	22.8	135.7
	60―100	1.35	7.80	12.50	0.11	2.10	1.44	13.2	105.6

### Experimental design

In this experiment, six treatments were designed and applied: (1) a flat plot (8 m × 4 m) with no basal fertilizer, no top dressing and no mulching (CK); (2) plastic film mulching with no basal fertilizer and no top dressing (MN_0_); (3) basal N (80 kg ha^−1^) and P (80 kg ha^−1^) [31] with no top dressing and no mulching (BN_1_); (4) plastic film mulching and basal N (80 kg ha^−1^) and P (80 kg ha^−1^) with no top dressing (MN_1_); (5) basal N (80 kg ha^−1^) and P (80 kg ha^−1^) and top dressing N (80 kg ha^−1^) with no mulching (BN_2_); and (6) plastic film mulching with basal N (80 kg ha^−1^) and P (80 kg ha^−1^) fertilizer and top dressing N (80 kg ha^−1^) (MN_2_).

**Fig 1:**
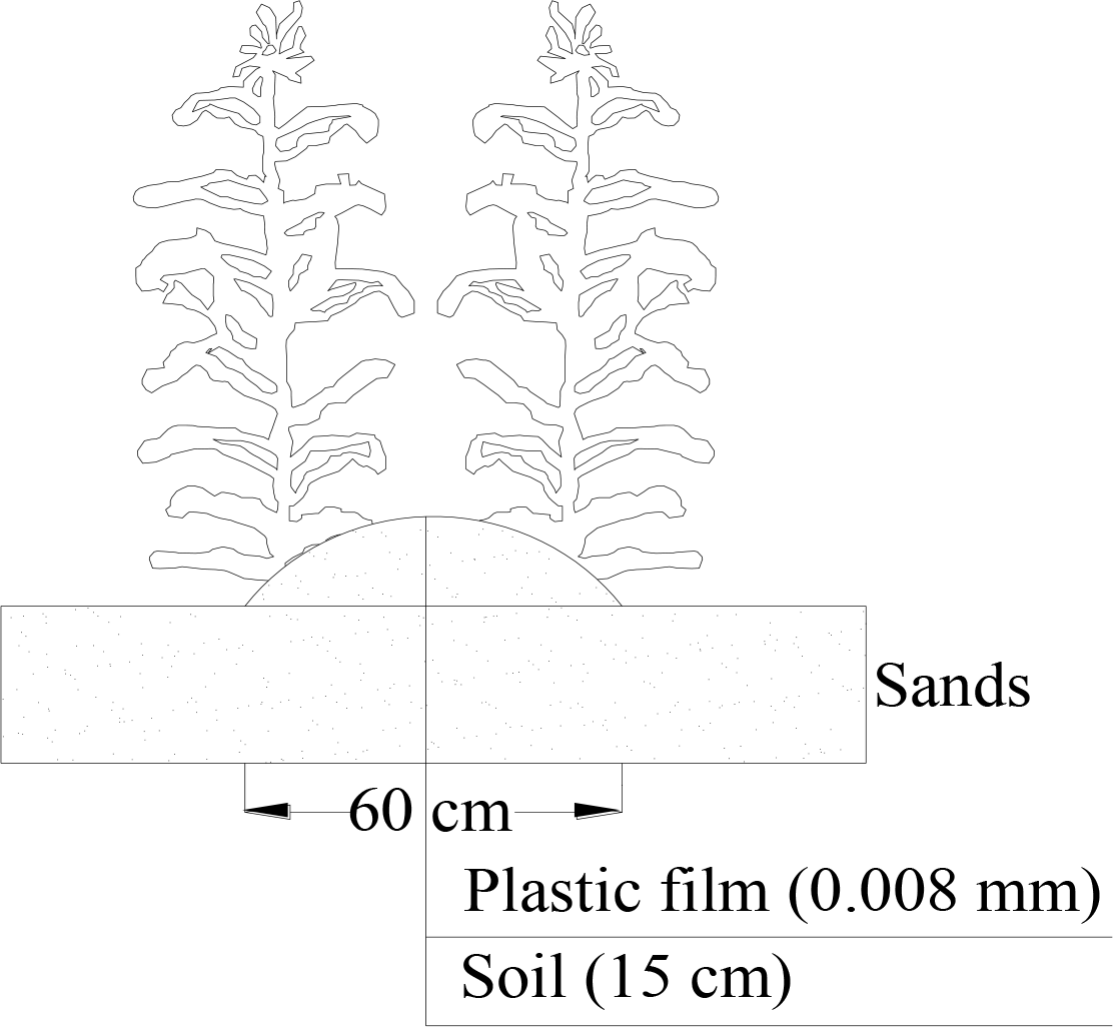
A sketch of the experimental arrangement system.

The experiment was laid out using a randomized block design with three replications; each plot was 8 m long and 4 m wide. The entire experimental area was ploughed and leveled each year during the three-year period over which the experiment was conducted. Following dividing and ridging of 18 experimental plots, basal fertilizers (80 kg N ha^−1^ and 80 kg P ha^−1^) were mixed in the soil for the BN_1_, MN_1_, BN_2_ and MN_2_ treatments. The basal fertilizers application schemes were chosen based on commonly practices used by local farmers. Maize was planted at a 30 cm row and 60 cm line spacing, and a sketch showing the width direction arrangement is presented in Fig 1. Mulching was laid over the soil surface layer of the ridges, 80 cm wide and 0.008 mm thick (Yonggu suye CO., LTD, Shaanxi, China).

The maize breed (*Zea mays* L., cv. ‘Liyu 18’) was sown on 21 April 2012 and 29 April 2013, using a hole-sowing tool (3-cm diameter). Top dressing N (80 kg ha^−1^) fertilizer was applied in late June (BN_2_, MN_2_). The maize crop was harvested on 18 September 2012 and 28 September 2013. After harvest, the plastic film was gathered and recycled by the manufacturer. Traditional tillage in dry farming areas of northern China involves mouldboard ploughing (motorized) to a depth of 16–18-cm, followed by a sequence of harrowing, smoothing, rolling and hoeing [17].

### Sampling and measurements

During the growing season, the soil water content (N = 3) was measured using the gravimetric method, and the depth interval spacing was 10 cm (from 0 to 100 cm). The soil water content was measured in the middle of the furrows, and the distance from a plant was 10 cm. The measurements were performed for nearly one month within the entire growth period (21 May, 28 June, 4 August and 9 September in 2012, and 19 May, 21 June, 26 July and 1 September in 2013, respectively). Such measurements were taken before rainfall. The soil water content measurements were performed at the same time as the measurements of the soil nitrate-N content [17].

The soil nitrate-N content (N = 3) was measured using a spectrophotometer (UV-VIS 8500Ⅱ, China), with sampling a depth interval of 10 cm, down to 100 cm, and the horizontal direction was three observation points, near of the plant 5 cm, move towards the furrow 30 cm and move towards the ridge 30 cm.

- First, 0.5 g of fresh soil was placed in a 100-mL triangular flask.
- Then, 50 ml of a 2-mol/L potassium chloride solution was added. The solution was shaken for half an hour until uniformity was reached.
- The solution was filtered, and 5 mL was placed in a spectrophotometer and examined at a wavelength of 210 nm [32].
- The nitrate content was determined using colorimetric analysis.

In this study, the soil nitrate-N below 100 cm soil depth and the ammonium-N throughout the whole soil profile will not be included in the nitrogen content measurements because most of the crop roots were mainly distributed in the soil depth of 0–100-cm [33].

### Grain yield and nitrogen balance estimation

The maize plants were sampled from a 4-m^2^ area in each plot at harvest for the measurements of grain yields and above ground biomass. Subsamples of grain and straw were oven-dried at 75°C for 24 h to calculate the moisture contents and dry matter. The total nitrogen content in grain and straw of the subsamples of both wheat and maize were determined by the micro-Kjeldahl method by digesting the sample in H_2_SO_4_– H_2_O_2_ solution [34]. Nitrogen uptake by plants was estimated by multiplying the grain and straw dry matter weight by their nitrogen concentrations [35].

**Fig 2:**
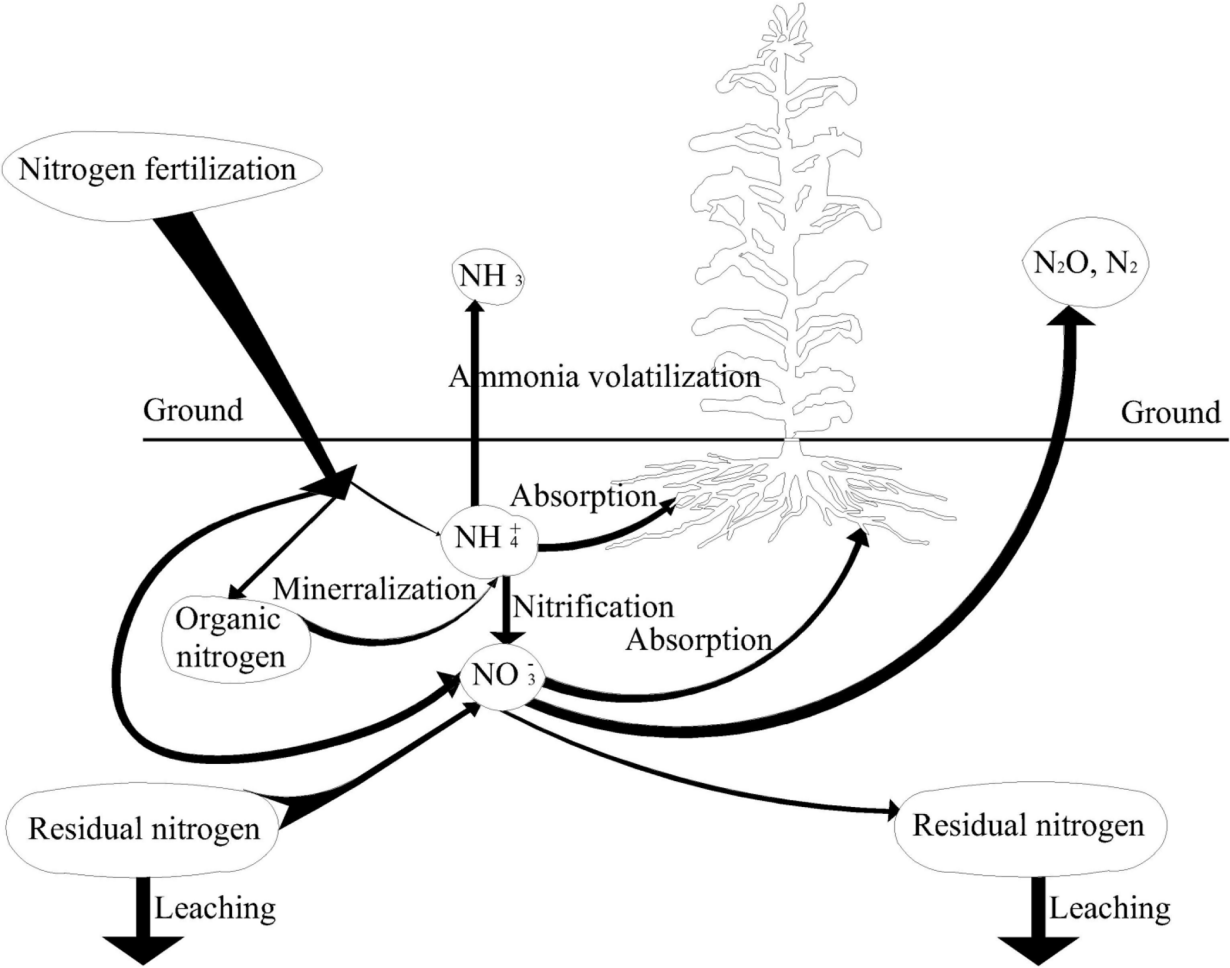
The fates of nitrogen fertilizer applied to the ordinary crop field.

The mass balance approach was used to assess the effect of biomass harvesting on nitrogen transport [36]. The amounts of nitrogen fertilizer application and soil nitrogen residual are the main source of soil nitrate-N. The soil nitrogen exported from the microcosm systems are mainly contained the amounts of nitrogen assimilated by plants, the amounts of nitrogen absorbed by the substrate, other losses, including ammonia volatilization, N_2_O and N_2_ emission [37, 38]. The fates of nitrogen fertilizer applied to the ordinary crop field are shown in Fig 2.

Items in the nitrogen balance were estimated in each plot for the two crop growing seasons from March to September in two consecutive years. For each period, the nitrogen balance can be calculated as following [35, 39].

$$N_{initial}+N_{input}+N_{min}=N_{uptake}+N_{residual}+N_{loss}\;(unit:\;kg\;N\;/\;ha)$$ (1)

- *N*_*initial*_ is initial soil nitrate-N in the 0–100 cm soil profiles. The initial of soil NO_3_^−^-N content was calculated as following.
  $$N_{initial},kg\;{ha}^{-1}=C_{1}(mg\;{kg}^{-1})\times h(cm)\times \rho (g\;{cm}^{-3})\times 10\times 0.01$$
          where *C*_1_ is soil nitrate-N content, h is soils thickness and ρ is soil bulk density.
- *N*_*input*_ is the nitrogen fertilizer application levels (0, 80, and 160 kg N ha^−1^).
- *N*_*min*_ is nitrogen mineralization.
- *N*_*uptake*_ is nitrogen uptake by plants.
- *N*_*residual*_ is soil nitrate-N residual in the 0–100 cm soil profiles.
- *N*_*loss*_ is soil nitrate-N loss in the 0–100 cm soil profiles.

*N*_*loss*_ is considered as mainly soil nitrate-N leaching since other nitrogen losses via volatilization, denitrification and erosion are relatively low under such environmental conditions [40–42].

Seasonal nitrogen mineralization (*N*_*min*_) was calculated by the balance of nitrogen fertilizer application levels and output in the control treatment (one control treatment with no basal fertilizer, no top dressing and no mulching (CK); another control treatment plastic film mulching with no basal fertilizer and no top dressing (MN_0_)) as following.

$$N_{min}=N_{uptake,\;0}+N_{residual,0}-N_{initial,0}\;(unit:\;kg\;N\;ha-1)$$ (2)

where *N*_*uptake*,0_, *N*_*residual*,0_ and *N*_*initial*,0_ are crop nitrogen uptake, residual and initial soil nitrate-N in the control treatment of 0–100 cm soil profile of the controls, respectively.Nitrogen recovery efficiency (*NRE*, in %) and nitrogen use efficiency (*NUE*, in %) were analyzed using the following equation [43, 44].

$$NRE=\frac{\left(N_{residual}-N_{initial})+(N_{uptake}-N_{uptake,0}\right)}{N_{input}}\times 100\%$$ (3)

$$NUE=\frac{N_{uptake.y}}{N_{input}}\times 100\%$$ (4)

where *N*_*uptake*.*y*_ is the nitrogen uptake contents in grain yield of maize (kg ha^−1^), *N*_*input*_ is nitrogen fertilizer application levels (kg ha^−1^).

The partial factor productivity of the fertilizer (*PFP*, in kg kg^−1^) was determined by the following equation [45–49].

$$PFP=\frac{Y}{F_{input}}$$ (5)

where *Y* is maize yield (kg ha^−1^), and F is the applied fertilizer (kg ha^−1^), i.e. the sum of nitrogen and phosphate fertilizer during each crop growing season.

### Statistical analysis

Analysis of variance was conducted on the soil water content, soil nitrate-N content and grain yield, which are using SAS 9.2 (SAS Institute Ltd., North Carolina, USA). Duncan’s multiple range test was used for paired mean comparisons at a 0.05 probability level [50].

## Results and Discussion

### Soil water status

**Fig 3:**
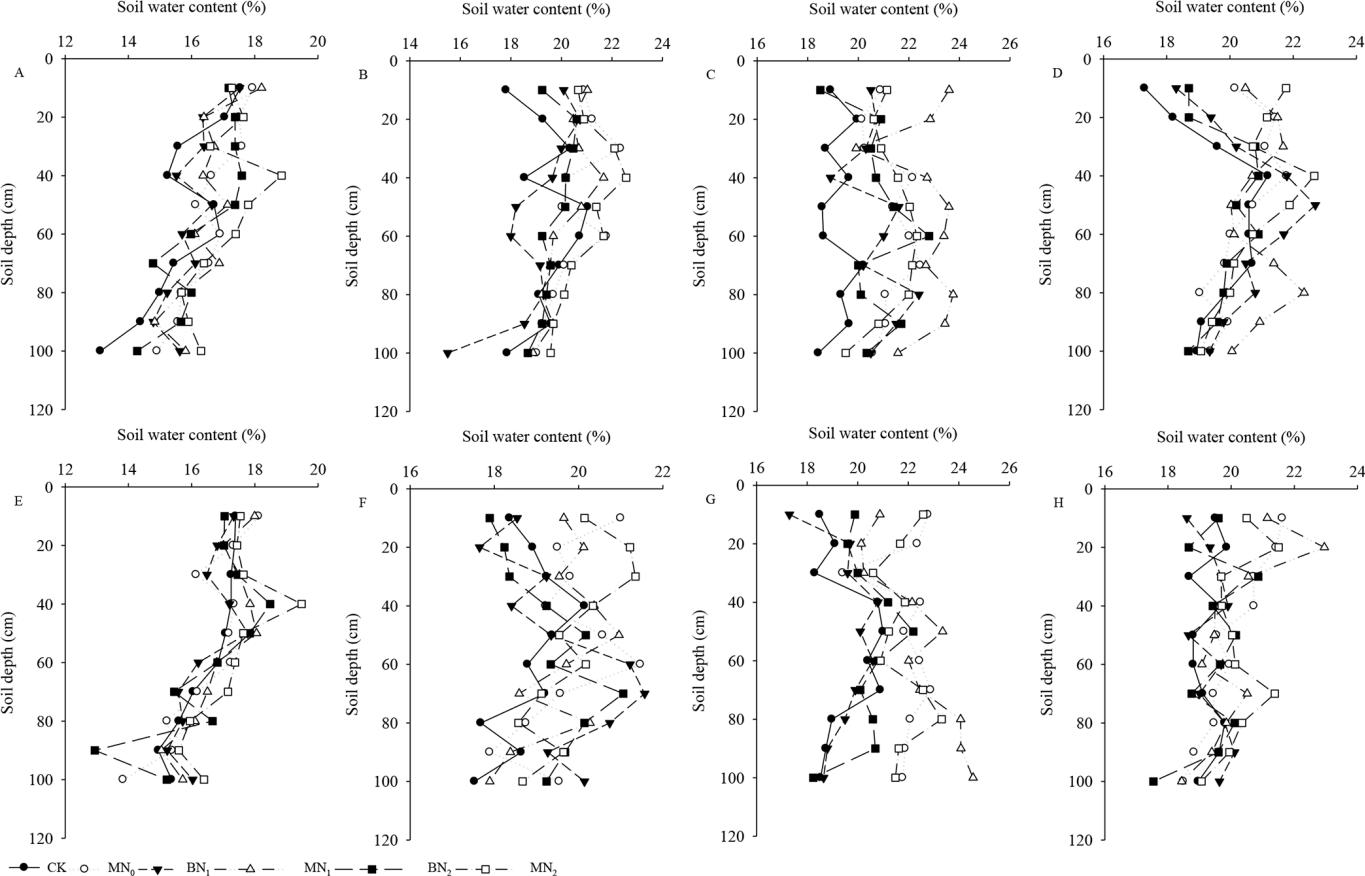
The effects of mulching and nitrogen fertilizer treatments on soil water contents in the study years.

The effects of mulching and nitrogen fertilizer treatments on soil water contents in two consecutive years are shown in Fig 3. In the top soil layers, the soil water content was a little high in the plastic film mulching than that without mulching. The mean soil water content from 0 to 40 cm without mulching were 3.35% and 2.03% lower than those measured in the corresponding mulching treatments in 31 days after sowing in 2012 and 20 days after sowing in 2013, respectively. Part of the reason may be that plastic film mulching was covered the ridge and furrow, which harvesting system makes better use of spat rain by collecting rainwater from the ridges, then improving water availability to crop growth compared with bare farming practices [5]. The difference of soil water content between mulching and no mulched treatment was significantly increased in 105 days after sowing in 2012 and 88 days after sowing in 2013, the mean soil water content of mulching treatment was 12.22% and 11.72% higher than without mulching. Compared with the CK treatment, the soil water content at harvest in both years was significantly higher in N_0_ than in N_1_ and N_2_ in the 40–100-cm soil layer, with no significant differences between nitrogen fertilizer treatments. In 68 days after sowing, all treatments under plastic film mulching not only significantly increased soil water content in 0–100-cm soil layer, but also regulated soil water distributed in the upper layer compared to their values on 31 days after sowing in 2012. The soil water content in the mulching treatment was higher than that of a bare plot at the time of seeding; after one month, however, these soil water contents were similar [51]. However, at harvest season of maize growth without mulch, the soil water content in all treatments in the 0–40-cm soil layer sharply decreased compared to values on 105 days after sowing in 2012.

The MN_1_ and MN_2_ treatments had the highest average soil water content at 0–40-cm at whole growing seasons, except 53 days after sowing in 2013. The result indicated that a higher soil water content merely exist in the surface layer under mulching treatment, which was probably due to lower surface run-off and evaporation because there was no change in surface soil porosity [52, 53]. The average soil water content (0–100 cm) in the CK treatment was 10.42%, 18.50% and 10.95% lower than that in the MN_0_, MN_1_ and MN_2_ treatments at 105 days after sowing in 2012, respectively, and 12.64%, 14.71% and 11.59% at 88 days after sowing in 2013. The same result was obtained that soil water content in the 20–100-cm layer decreased steadily in all of the treatments, and the soil water content of the mulched treatments were always significantly (*p* < 0.05) higher than that of CK [54]. The mulching of the ridge and furrow regulated the soil water conditions, which caused the soil water content of the various soil layers to differ from each other. However, the average soil water content in the CK treatment was only 3.70%, 6.69% and 5.82% lower than that in the MN_0_, MN_1_ and MN_2_ treatments at 134 days after sowing in 2012, respectively, and 3.84%, 4.20% and 4.93% at 125 days after sowing in 2013. The result may be related to the canopy density of crop plants at harvest, and the effect of mulching on soil water contents was decrease with the increase of growth period. Based on the above results, we can draw a conclusion that plastic film mulching adjusts the regularity of the vertical distribution of the soil water is limited. These results are compatible to those of previous studies. The soil water content was significantly decreased in the mulching treatment compared with no-mulching at the same fertilizer in June, and there was no significant difference in September [17]. The soil water content between mulching and no-mulching treatment variations were reduced from mid-June to September, most likely because of the more robust canopy shading at the late growth stage [1, 55, 56].

### Soil nitrate-N content

**Fig 4:**
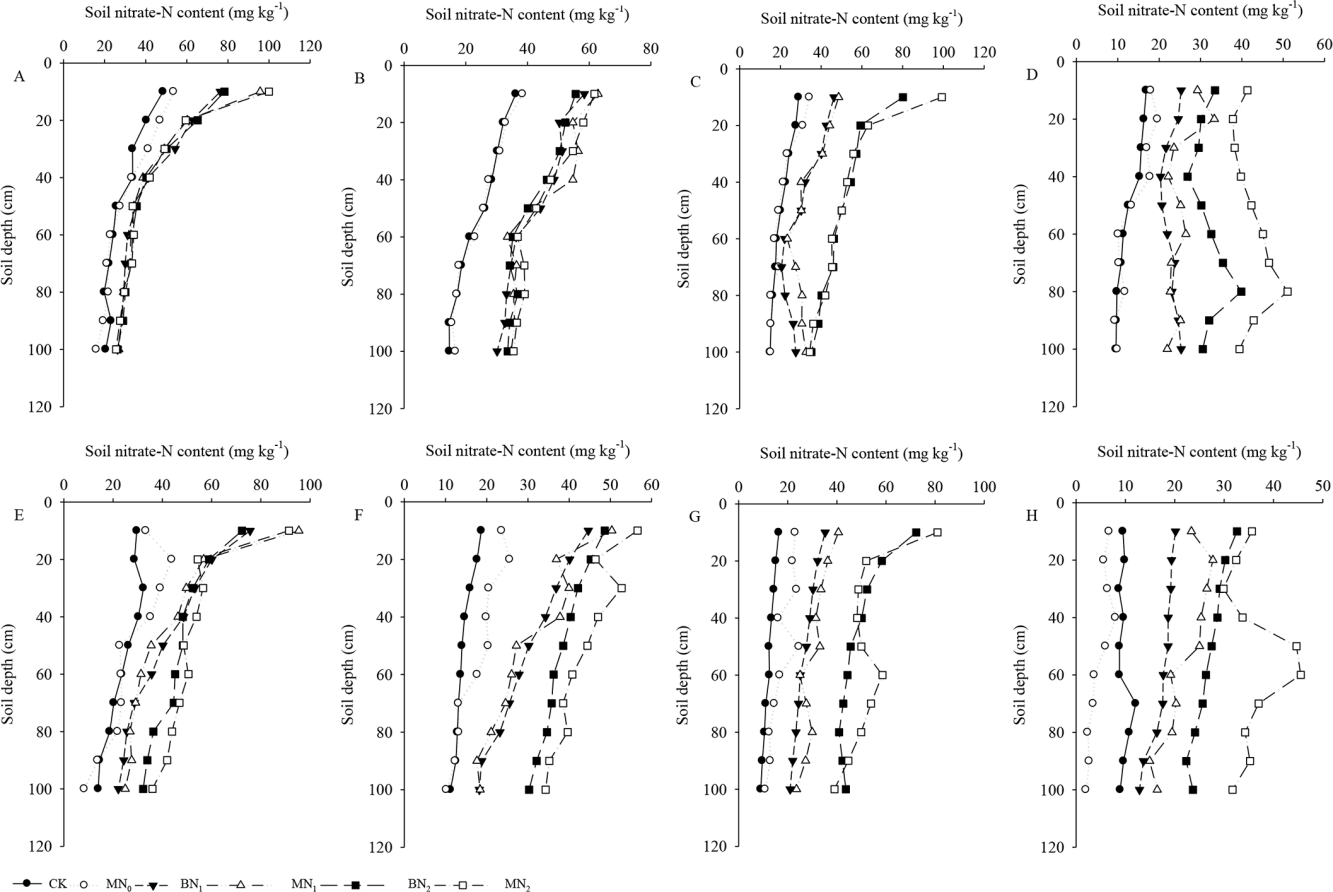
The effects of mulching and nitrogen fertilizer level on soil nitrate-N content in two consecutive growing seasons.

The effects of mulching and nitrogen fertilizer level on soil nitrate-N content in two consecutive growing seasons are shown in Fig 4. The soil nitrate-N content was significantly decreased with increase of the soil depth under basal nitrogen fertilizer treatments in 31 days after sowing in 2012 (Fig 4A). In BN_1_, MN_1_, BN_2_ and MN_2_ treatments, the mean soil nitrate-N content in top soil layer (0–50-cm) was 46.04%, 46.22%, 43.63% and 47.15% higher than the upper soil layer (50–100-cm). Well, there are two reasons, one because the basal fertilizer was mainly in the surface layer due to low rainfall [57], and the other reason is that the soil organic matter content in the surface layer resulted from nitrate-N via digestive functions [58]. The nitrogen fertilizer treatment significantly increased the soil nitrate-N content in the early growth stage compared with the CK treatment. However, there was no significant difference between mulching and no mulched treatment at the same nitrogen fertilizer levels. The gap between the nitrogen fertilizer and no fertilizer treatment was significantly increased in 68 days after sowing in 2012 (Fig 4B). The mean soil nitrate-N content in CK was 42.90% and 42.78% lower than BN_1_ and BN_2_ in mulching treatment, and the mean soil nitrate-N content in MN_0_ was 44.95% and 45.65% lower than MN_1_ and MN_2_ in no mulch treatment. The plastic film mulching treatment was particularly effective when compared with the nitrogen fertilizer application treatment. The mean soil nitrate-N content in CK was only 2.32% lower than MN_0_ treatment, and the BN_2_ was 7.23% lower than MN_2_ treatment. The same result was discovered that the ability of the plastic film mulching to improve the soil water content, meanwhile, the availability nitrogen was improved with mulching [59].

The nitrogen fertilizer applications significantly affect the soil nitrate-N content in the soil profile of 0–60-cm in 105 days after sowing in 2012, and the soil nitrate-N content in topdressing nitrogen treatments was found a high concentration in top soil layers of 0–20-cm (Fig 4C). The mulching treatment increased the soil nitrate-N content was only observed in the 0–40-cm soil layers. Compared with the CK treatment, the soil nitrate-N content was significantly lower in N_0_ than in N_1_ and N_2_ with mulching treatment in the 0–100-cm soil layer, and the mean soil nitrate-N content in CK was 39.12% and 60.67% lower than MN_1_ and MN_2_ treatment. However, there was no significant difference in soil nitrate-N content at the same levels of nitrogen fertilizer application. The soil nitrate-N content was increased with the increase of soil depth from 20 cm to 80 cm in 134 days after sowing in 2012 (Fig 4D). The reason may be the higher nitrogen application rate have exceeded the nitrogen uptake by plants and contributed to soil nitrate-N accumulation in the 40–100-cm soil layer [60, 61].

In the basal and top dressing treatments, the mean soil nitrate-N content in plastic film mulching treatment was 24.41% higher than without mulch treatment in 0–100-cm soil layer, but the basal and no top dressing was 8.45% higher than without mulch treatment and no fertilizer treatment was 6.63% higher than without mulch treatment. The lowest soil nitrate-N content in CK treatment, which was 44.71%, 49.39%, 60.13% and 69.86% lower than BN_1_, MN_1_, BN_2_ and MN_2_ treatments, respectively. The soil nitrate-N content in 0–100-cm soil layer had the same trend of change law in the whole growth period in 2013. The only difference is that the soil nitrate-N content was not increased with the increase of soil depth from 20 cm to 80 cm in 125 days after sowing in 2013, except the MN_2_ treatment. The similar results was reported that soil moisture had a significant influence on soil nitrate-N movement in the 0–100-cm soil profiles [62]. The content of soil nitrate-N in the 20–80-cm soil layers was only slightly increased in the treatments of 200 kg N ha^−1^ [35, 63].

### Changes in nitrate-N in the soil profiles

**Fig 5:**
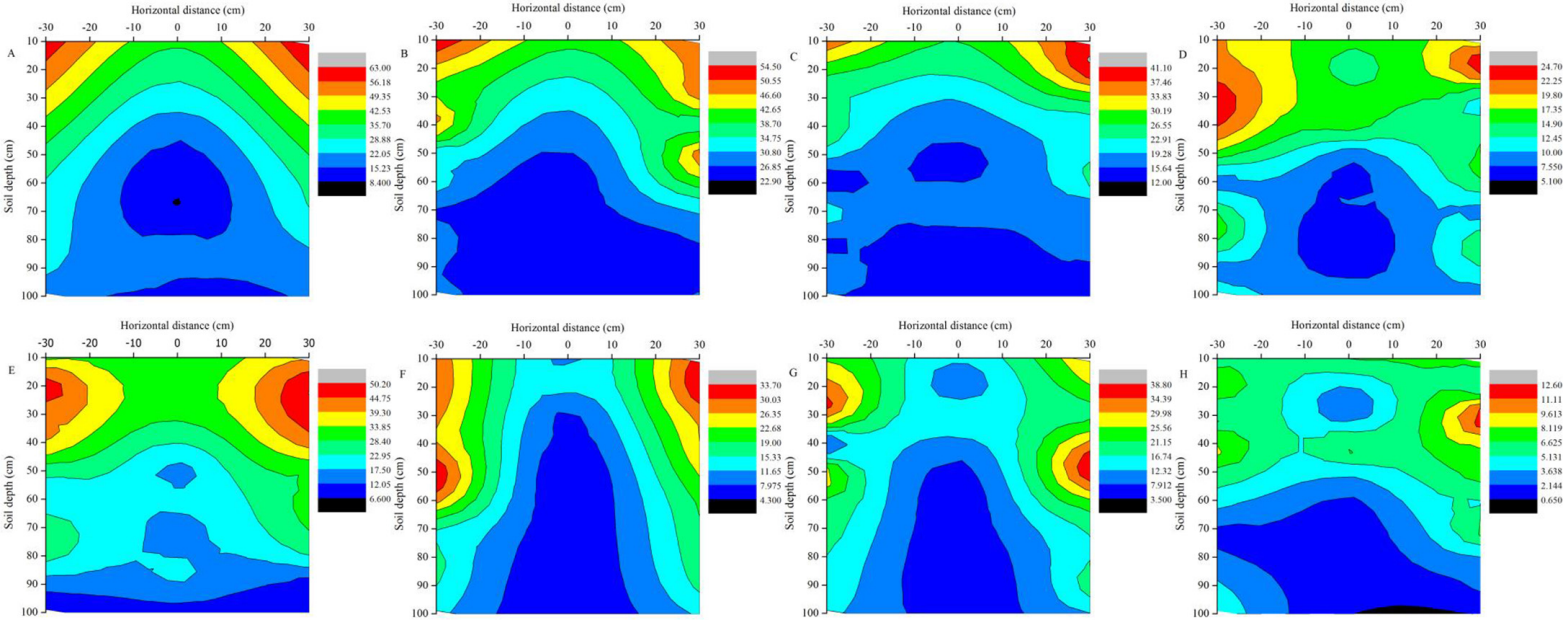
The effects of mulching and no-fertilizer on the vertical distribution of nitrate-N content in two consecutive growing seasons (mg kg^−1^). 2012: (A) 31 days after sowing, (B) 68 days after sowing, (C) 105 days after sowing and (D) 134 days after sowing; 2013: (E) 20 days after sowing, (F) 53 days after sowing, (G) 88 days after sowing and (H) 125 days after sowing.

The dynamics of the soil nitrate-N in the root region area of the no-fertilizer treatment with plastic film mulching in 2012 and 2013 are shown in Fig 5. Soil nitrate-N in the 0–100-cm profiles ranged from 8.4 mg kg^−1^ to 63.0 mg kg^−1^ during the 31 days after sowing in 2012 and was similar in 2013 (Fig 5A and 5E). The soil nitrate-N concentration was consistently decreasing with growing stages and the content of soil nitrate-N was little higher in both sides of root system. The results indicate that mean nitrate-N concentration in 0–100-cm profiles at 31 days after sowing was 2.22 times higher than the mean nitrate-N concentration at harvest time. In the current experiments, soil nitrate-N in the 0–100-cm soil layer ranged from 5.1 mg kg^−1^ to 24.7 mg kg^−1^ at harvest in 2012 and from 0.65 mg kg^−1^ to 12.6 mg kg^−1^ in 2013 (Fig 5D and 5H). There was a large horizontal difference in the soil nitrate-N concentration in the top layers (0–40-cm) about a month after sowing in two consecutive years. The trend in the soil nitrate-N content distribution exhibited symmetrical shapes along the center of the furrow.

**Fig 6:**
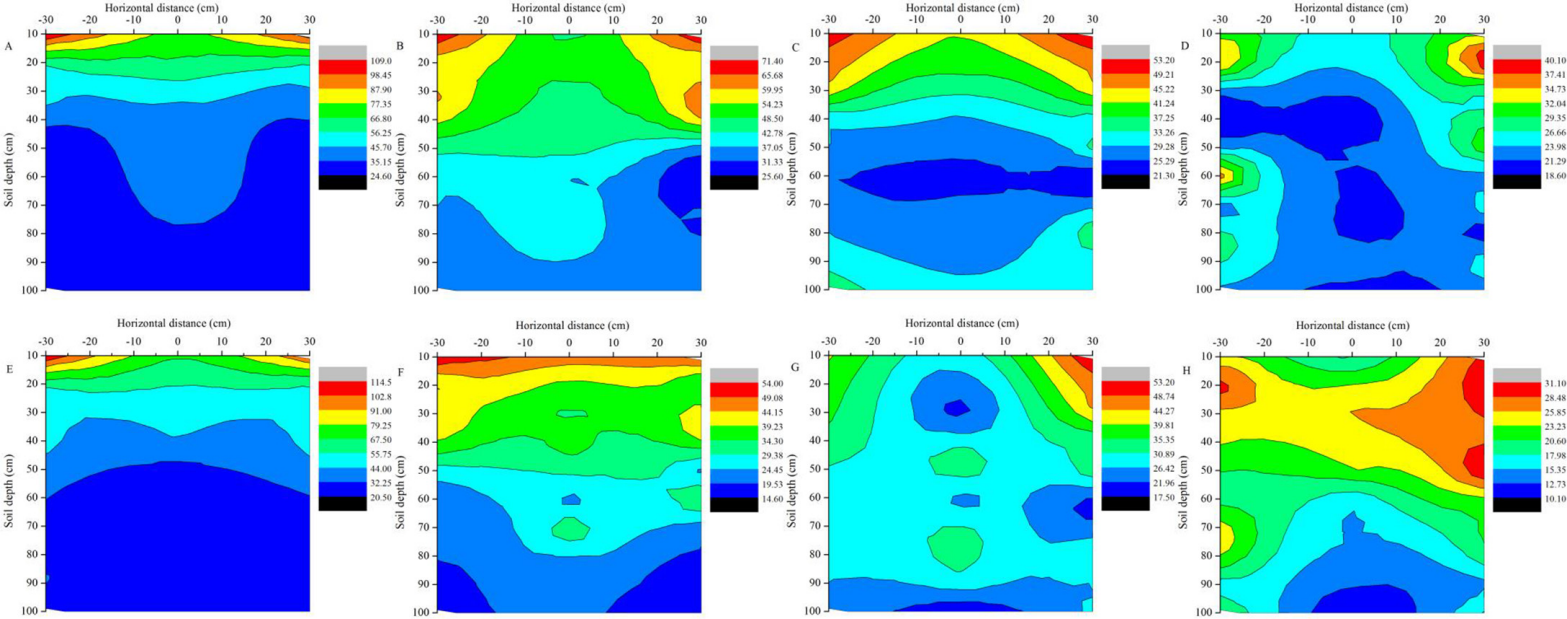
The effects of mulching and basal-fertilizer on the vertical distribution of nitrate-N content in two consecutive growing seasons (mg kg^−1^). 2012: (A) 31 days after sowing, (B) 68 days after sowing, (C) 105 days after sowing and (D) 134 days after sowing; 2013: (E) 20 days after sowing, (F) 53 days after sowing, (G) 88 days after sowing and (H) 125 days after sowing.

The effects of mulching and basal fertilizer application on soil nitrate-N concentration in the soil profiles are shown in Fig 6. The vertical distribution of soil nitrate-N was similar to the treatment without nitrogen fertilizer at 31 days after sowing, but the contents of soil nitrate-N in basal fertilizer treatment was significantly higher than that without fertilizer treatment (Fig 6A). The mean soil nitrate-N concentration in the 0–100-cm soil layer in MN_1_ was 28.91% higher than MN_0_ treatment. In the whole growth period, the soil nitrate-N concentration was also sustained downward trend. The soil nitrate-N in the top soil layers was move downward to the soil layer of 60–90-cm in 68 days after sowing in 2012 (Fig 6B). In 105 days after sowing, the standard symmetrical distribution reduced gradually with the soil depth, but persisted under the plastic film mulching conditions (Fig 6C). The soil nitrate-N concentration in the root absorption area was lower than in the other areas, and the trend gradually increased with the advancement of reproductive period. The soil nitrate-N in 0–30-cm layers was generally higher in the earlier stage of maize growth than later period. At harvest, the soil nitrate-N concentration was significantly decreased in the whole soil profiles, and the content of soil nitrate-N was ranged from 18.6 mg kg^−1^ to 40.1 mg kg^−1^ in 2012 and from 10.1 mg kg^−1^ to 31.1 mg kg^−1^ in 2013 (Fig 6D and 6H).

**Fig 7:**
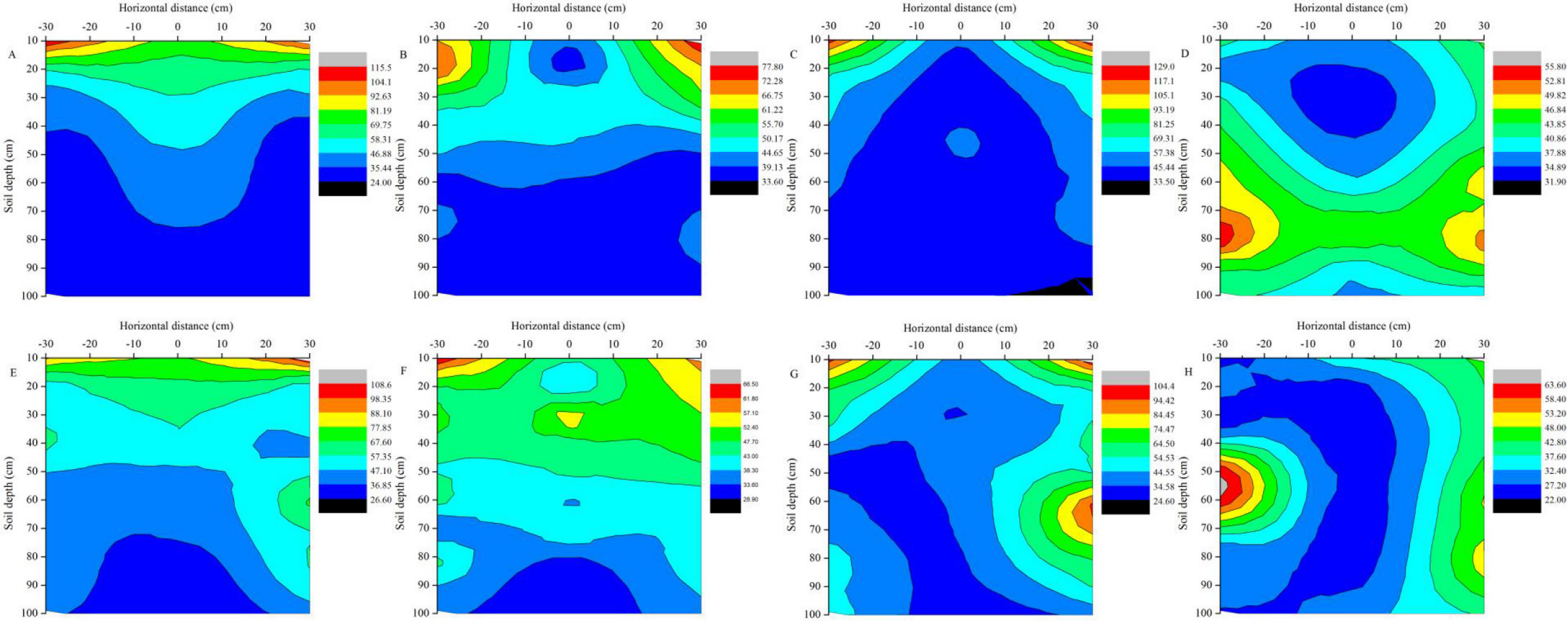
The effects of mulching and basal-topdressing-fertilizer on the vertical distribution of nitrate-N content in two consecutive growing seasons (mg kg^−1^). 2012: (A) 31 days after sowing, (B) 68 days after sowing, (C) 105 days after sowing and (D) 134 days after sowing; 2013: (E) 20 days after sowing, (F) 53 days after sowing, (G) 88 days after sowing and (H) 125 days after sowing.

The vertical distribution of the soil nitrate-N in root area of the basal-topdressing-fertilizer treatment with plastic film mulching in 2012 and 2013 are shown in Fig 7. The results indicate that high contents of soil nitrate-N were mainly distributed at 0–20-cm at 31 days after sowing, and the soil nitrate-N concentration in the basal-topdressing-fertilizer treatment was 1.58 times higher than that did not receive fertilizer (Fig 7A). The mean soil nitrate-N content was ranged from 25.6 mg kg^−1^ to 100.0 mg kg^−1^ at 31 days after sowing in 2012. The nitrate-N in the root zone was reduced in the soils of the basal and top dressing treatments with the plastic film mulching at 68 and 53 days after sowing in 2012 and 2013, respectively. There were significantly soil nitrate-N concentration differences in the top soil layers (0–40-cm), but there was no significant difference in the soil nitrate-N content below 50 cm, and the symmetrical distribution was the same as that observed in the MN_1_ treatment in the top layers (0–40-cm) after topdressing treatment. The mean soil nitrate-N content in the topsoil (0–40-cm) was reduced to 39.31 mg kg^−1^ from 67.69 mg kg^−1^ at 134 days after sowing in 2012. The soil nitrate-N content in the subsoil (60–100-cm) increased at 105 and 88 days after sowing compared with 68 and 53 days after sowing in 2012 and 2013, respectively. Especially, there was a nitrate-N accumulation area coming close to 70 cm in 2012 and 60 cm in 2013 at harvest time.

Overall, the mean soil nitrate-N content with basal-fertilizer treatment increased to approximately 13.44% at 31 days after sowing compared with no-fertilizer treatment. In addition, the basal-topdressing-fertilizer treatment greatly increased the soil nitrate-N content in the upper layer of soil (0–40-cm), and the mean soil nitrate-N content was increased nearly 50 mg kg^−1^ at 105 days after sowing compared with no-fertilizer treatment. The soil nitrate-N was concentrated at 60 cm to 80 cm at harvest time in top dressing treatment, and there was no difference along the entire soil profile in all treatment. Previous studies have suggested that the amount of soil nitrate-N content in the topsoil (0–50-cm) was significantly different from that in the subsoil (50–100-cm), and the subsoil nitrate residues were well correlated with root intensity [64]. We found that the nitrate-N concentration in the root absorption area was lower than in the other areas.

### Soil nitrate-N leaching and balance estimation

The soil nitrate-N leached varied considerably between the different plastic film mulching and fertilization treatments, ranging from 0 kg ha^−1^ to 55.32 kg ha^−1^ in 2012 and from 0 kg ha^−1^ to 70.11 kg ha^−1^ in 2013 (Table 2). There was a significantly increase in soil nitrate-N leaching with the increase of nitrogen fertilizer application rate. The plastic film mulching has a fine effect on preventing the soil nitrate-N leaching from top to upper soil layers. The soil nitrate-N leaching amount in mulching treatment was 28.61% and 39.14% lower than no-mulching in basal-fertilizer treatment in both years, and the mulch effect attained to 42.55% and 65.27% in basal-topdressing-fertilizer application. These previous studies reported that the application of higher levels of nitrogen resulted in higher soil nitrate-N leaching from the soil, greatly exceeding the crop requirement and resulting in lower fertilizer use efficiency [27, 65].

**Table 2:** Soil starting Nitrogen (N_initial_), nitrogen input (N_input_), nitrogen mineralization (N_min_), nitrogen uptake by plants (N_uptake_), soil nitrate-N residual (N_residual_) in the 0–100-cm profiles and nitrogen leaching (N_loss_) below 100 cm depth as affected by mulching and nitrogen fertilizer application rate in the maize growing system in 2012 and 2013 (kg ha^−1^).

Treatment		N_initial_	N_input_	N_min_	N_uptake_	N_residual_	N_loss_
2012	CK	66.34	0	20.22	56.23±2.94e	30.32±2.15d	–
	MN_0_	66.34	0	20.70	52.36±0.79e	34.67±1.81d	–
	BN_1_	66.34	80	20.22	75.65±2.2d	61.25±2.02c	29.67±4.21b
	MN_1_	66.34	80	20.70	80.63±2.89c	64.75±2.19c	21.18±3.68c
	BN_2_	66.34	160	20.22	105.27±4.04b	85.97±3.6b	55.32±7.62a
	MN_2_	66.34	160	20.70	112.72±1.84a	102.05±6.54a	31.78±4.72b
	Correlation coefficient (*r*)						
	N_input_ × N_uptake_				0.9895		
	N_input_ × N_residual_					0.9814	
	N_input_ × N_loss_						0.7155
2013	CK	56.85	0	18.82	52.95±1.35d	22.74±2.78d	–
	MN_0_	56.85	0	13.11	52.35±1.37d	17.63±4.92d	–
	BN_1_	56.85	80	18.82	69.38±3.78c	46.02±3.69c	40.29±3.41b
	MN_1_	56.85	80	13.11	72.55±4.88c	52.91±3.54c	24.52±8.37c
	BN_2_	56.85	160	18.82	98.20±9.76b	67.37±7.67b	70.11±15.84a
	MN_2_	56.85	160	13.11	109.25±1.63a	96.38±4.59a	24.35±4.88c
	Correlation coefficient (*r*)						
	N_input_ × N_uptake_				0.9749		
	N_input_ × N_residual_					0.9445	
	N_input_ × N_loss_						0.3975

Means within columns followed by the same lowercase letters are not significantly different (*p* < 0.05) according to Duncan’s multiple range tests for irrigation treatments within same season.

The nitrogen fertilizer application levels was significantly affect the nitrate-N residual amount, and the mulching was only obviously effect the nitrogen residual amount in basal-topdressing-fertilizer treatment in two consecutive years. The highest nitrogen residual was obtained in MN_2_ treatment, which was 15.75%, 36.55%, 39.88%, 66.02% and 70.29% higher than BN_2_, MN_1_, BN_1_, MN_0_ and CK treatment in 2012, and the similar result was observed in 2013. For this reason, many scholars have conducted a lot of research, residual nitrate-N increases quickly when the nitrogen fertilizer application rate exceeds a certain value, but that corresponding grain yield do not increase significantly after this certain value is reached [66, 67]. The variation tendency of nitrogen uptake was similar to nitrogen residual, but the single factors of mulch or nitrogen fertilizer were significant for nitrogen uptake amount. Surprisingly, the nitrogen uptake amount in mulching treatment was 6.88% lower than that without mulch treatment in 2012. However, the MN_1_ treatment was 6.58% higher than BN_1_ treatment and the MN_2_ treatment was 7.08% higher than BN_2_ treatment in 2012, and the result was similar as 2013.

### Grain yield and nitrogen use efficiency

The effect of mulching, basal fertilizer and top dressing nitrogen fertilizer on grain yield, dry matter accumulation, Nitrogen recovery efficiency (NRE), nitrogen use efficiency (NUE) and the partial factor productivity of the fertilizer (PFP) are shown in Table 3. The result indicated that higher grain yield of maize was observed in CK treatment compared with the MN_0_ treatment, but there was no significant difference between the mulching and no-mulching treatments under no basal fertilizer or top dressing. However, there was a significant difference for the plastic film mulching treatment with basal fertilizer or top dressing, and plastic film mulching had a positive effect on grain yield. The yield increased with an increase in the basal fertilizer and plastic film mulching, and the grain yield in MN_1_ treatment increase 5.27% and 16.18% compared with BN_1_ treatment in both years. Further, the MN_2_ treatment was 19.46% and 18.40% higher than BN_2_ treatment in 2012 and 2013, respectively. The yield increased with an increase in the basal fertilizer, top dressing and plastic film mulching, and the grain yield increase ranged from 31.41% to 79.06% compared with the CK treatment in 2012 and from 33.30% to 83.61% in 2013. Other studies found that film mulching in field experiments increased the maize grain yield by approximately 20–30% in very wet years, 60–95% in average and drought years [68]. Additional mulch in furrows increased the maize grain yield by 8–25% in the semi-arid Loess region of northwestern China [69].

**Table 3:** The effects of mulching, basal fertilizer and top dressing nitrogen fertilizer on grain yield, dry matter accumulation, Nitrogen recovery efficiency (NRE), nitrogen use efficiency (NUE) and the partial factor productivity of the fertilizer (PFP) in the study years.

Treatment		GY (Mg ha^−1^)	DM (Mg ha^−1^)	NRE (%)	NUE (%)	PFP (kg kg^−1^)
2012	CK	3.75±0.33d	10.12±0.53e	–	–	–
	MN_0_	3.33±0.06d	9.43±0.14e	–	–	–
	BN_1_	4.93±0.39c	12.48±0.36d	17.91±5.26	28.12±1.47	30.84±2.42
	MN_1_	5.19±0.09bc	13.3±0.48c	33.35±4.60	29.45±0.45	32.46±0.54
	BN_2_	5.63±0.14b	15.79±0.61b	42.92±4.67	18.91±0.55	23.45±0.60
	MN_2_	6.72±0.36a	16.91±0.28a	60.05±2.95	20.16±0.72	28.01±1.51
2013	CK	3.57±0.24d	9.8±0.25d	–	–	–
	MN_0_	3.24±0.28d	9.68±0.25d	–	–	–
	BN_1_	4.76±0.07c	11.79±0.64c	6.97±4.26	26.48±0.67	29.77±0.44
	MN_1_	5.53±0.24b	12.33±0.83c	20.30±10.46	29.45±0.77	34.58±1.47
	BN_2_	5.54±0.36b	13.75±1.37b	34.85±9.90	17.34±0.95	23.09±1.49
	MN_2_	6.56±0.08a	15.29±0.23a	60.25±3.05	18.14±1.22	27.34±0.34

Means within columns followed by the same lowercase letters are not significantly different (*p* < 0.05) according to Duncan’s multiple range tests for irrigation treatments within same season.

The dry matter accumulation of maize was increased with the increase of nitrogen fertilizer application rate, and the plastic film mulching was significantly increased the dry matter accumulation under basal, top dressing fertilizer conditions. Compared to CK treatment, the dry matter accumulation was 23.31%, 31.43%, 55.99% and 67.05% higher in BN_1_, MN_1_, BN_2_ and MN_2_ treatment in 2012, and the result was similar as 2013. However, the dry matter accumulation in MN_0_ treatment was 6.88% lower than CK treatment in 2012.

The nitrogen recovery efficiency was increased with the increase of nitrogen fertilizer application rate and plastic film mulching. The nitrogen recovery efficiency was ranged from 17.91% to 60.05% in 2012 and from 6.97% to 60.25% in 2013. In two consecutive years, the highest nitrogen recovery efficiency was also observed in MN_2_ treatment. However, the BN_2_ treatment was only 42.92% and 34.85%, which was significantly lower than MN_2_ treatment. The result indicated that the mulching was an obvious increase in nitrogen recovery efficiency with nitrogen application. In 2012, the nitrogen use efficiency and partial factor productivity of the fertilizer did not significantly differ between treatments. The more nitrogen fertilizer application, the lower values of nitrogen use efficiency and partial factor productivity of the fertilizer was obtained in both year.

## Conclusions

In the topsoil layer, the soil water content in the mulching treatment was significantly higher than that without mulching treatment. High soil nitrate-N content was mainly distributed in the 0–40-cm soil layers with basal fertilizer, and the soil nitrate-N was concentrated at 60 cm to 80 cm at harvest time in top dressing treatment. The yield increased with an increase in the basal fertilizer, top dressing and plastic film mulching, and the grain yield increase ranged from 31.41% to 83.61% in two consecutive years. The MN_1_ and MN_2_ treatment is recommended because it increased the grain yield and improved the fertilizer use efficiency, compared with the no-mulching treatment.

## Supporting Information

S1 File Data of mean soil water contents in both years. (XLS)

S2 File Data of mean soil nitrate-N contents in both years. (XLS)
